# Neoliberalism in Latin America: effects on health system reforms

**DOI:** 10.11606/s1518-8787.2020054001806

**Published:** 2020-07-20

**Authors:** Leila Bernarda Donato Göttems, Maria de Lourdes Rollemberg Mollo

**Affiliations:** I Universidade de Brasília Faculdade de Economia, Administração, Contabilidade e Gestão de Políticas Públicas Programa de Pós-Graduação em Economia BrasíliaDF Brasil Universidade de Brasília. Faculdade de Economia, Administração, Contabilidade e Gestão de Políticas Públicas. Programa de Pós-Graduação em Economia. Brasília, DF, Brasil

**Keywords:** Health Systems, Health Care Reform, Health Policy, Latin America

## Abstract

**OBJECTIVE:**

To analyze the recommendations of international organizations based on the Washington Consensus on health system reforms of selected countries in Latin America and the Caribbean in the 1980s and 1990s and to investigate the effects of the competitive market logic on public action in the health system.

**METHODS:**

Comparative analysis of the characteristics of health system reforms conducted in the 1980s and 1990s, still seen in Brazil, Argentina, Chile, Colombia, Mexico and Peru. Data were collected by documental analysis and literature review. The systems were described based on the characteristics of: co-payment, privatization mechanisms, decentralization, fragmentation of the system, integration of funding sources and coverage of the population (universal or segmented).

**RESULTS:**

The reforms were implemented differently, worsening inequalities in health service delivery systems. Changes related to the neoliberal idea of transforming public action in the direction of private logic point to the predominance of competition rules and the reduction in economic costs in all countries analyzed, contrary to the logic of universal health systems.

**CONCLUSION:**

The reduction in economic costs, the fragmentation of systems and inequalities in the provision of health services, among others, may mean other future costs resulting from low protection to the population’s health. A striking and multidimensional counter-reform is essential to make health a right of all again, in a solidarity system that can lead to the reduction in inequalities and a more democratic society.

## INTRODUCTION

This article analyzes the effects of neoliberal recommendations of international organizations on the development of health systems in Latin America and the Caribbean (LAC). These recommendations have characterized neoliberalism around the world since the late 1970s. In LAC, this occurred since the 1980s, with the exception of Chile, whose liberalization began with the Pinochet dictatorship in the 1970s.

This is the period of the “debt crisis”, resulting from the increase in US interest rates in 1979. This led LAC countries to default on loans, starting with Mexico, spreading a wave of financial shock across the continent^1^. In October 1983, 27 low- and middle-income countries, many in the Americas, were defaulting on their loans or in the process of rescheduling debts^3,6^.

In this period, the International Monetary Fund (IMF) and the World Bank (WB) provided loans to debtor countries to alleviate balance of payments deficits and the burden of debt service, as well as to bail out the private banking sector in high-income countries^6,8^. Economic measures to open domestic markets to foreign penetration and stimulate low-cost exports were associated with these loans. These recommendations were based on the Washington Consensus, which contained ten measures, from fiscal discipline, reduction in public spending, tax reform (increasing the tax base and taxing mainly indirectly), to commercial and economic opening of countries, liberalization of the exchange rate and foreign trade, elimination of restrictions on direct financial investment, privatization and sale of state-owned enterprises, deregulation and intellectual property rights^1^. These measures led to the reduction in the welfare state and the globalization of capital interests, adopted primarily in Margaret Thatcher’s Britain and Ronald Reagan’s US, and, with the debt crisis, spread rapidly to Third World economies such as LAC^1^.

In Latin America, a type of late capitalism developed with an accelerated and disorderly urbanization process and an increase in informal activities, in which there are abundant servile works and impregnated by relationships of subordination and low protection^10^. The implementation of the welfare state was late, incomplete and quite unequal among countries. This fact, associated with the fragile citizenship of the population, caused, at the time of the debt crisis, the “reforms” to aggravate the institutional segmentation and operational fragmentation of social protection systems, increasing the inequalities of coverage and access of health systems in the region^11^.

The changes led to reconfiguration of health systems and were unequally and gradually adopted by LAC countries in the 1980s and 1990s. They were based on the separation between social and economic policy, combining short-term compensation programs with renewed confidence in economic growth and the spill effect in the long run, relegating the conflict around production and appropriation of wealth to the background^6^. In the first decade of the 21st century, in the period known as the “pink tide” of LAC, many countries succeeded in reducing extreme poverty and social inequalities, with increased income and formal jobs, fiscal adjustments, increased social spending and tax reforms^11^. However, a system of social protection based on the right of universal access has not been developed, and this right is currently suffering from the effects of an acute resurgence of neoliberalism.

The origins of neoliberalism date back to the creation of a theoretical framework for regulating life in society, the individual and the State in such a way as to oppose the planning states and with powers to interfere in individual freedom, property rights and free exchanges in the market. It is based on the systematic use of state power, under the ideological appearance of “non-intervention,” to impose a hegemonic project of recomposition of the capital rule at five levels: allocation of domestic resources, international economic integration, state reproduction, ideology and reproduction of the working class^7,9,13^.

Neoliberalism causes important changes in the conformation of social policies, especially those resulting from the reconfiguration of states with deregulation and privatizations, which remove them from various areas and reduce their performance^7,9,13^. The new global standard requires administrative and social devices to cost less and to focus on the demands of economic competition. Themes such as management efficiency and new methods to provide services to the population emerge in public policies. This thought feeds the will to impose, at the heart of public action, values, practices and the functioning of a private company that lead to the establishment of a new government practice – flexible, reactive, market-based and consumer-oriented state – the management^15^. Thus, it respect an economic logic, which prioritizes cost reduction, the notion of equality in receiving the health service seeks to ensure the basics in a focused way and the standard of conduct not only of the company, but also of the individual and of the State, becomes the standard of competition, typical of the market^6,8,12,15^.

The new genre of social policy then consists of weakening the bargaining power of trade unions, degrading labor law, lowering the cost of labor, reducing the value of pensions and the quality of social protection, in the name of adapting to globalization^13^. However, it is not the end of the State, but the relativization of its role as an integrating entity of all dimensions of collective life, since they tend to delegate much of their functions to private companies that are often already globalized or obey world norms. The State puts itself at the service of specific oligopolistic interests and does not hesitate to delegate to them a considerable part of the sanitary, educational, tourist and recreational management of the population. In other words, the general rule in neoliberalism is competition and, with it, the “compression of the wage costs and social protection expenditures of states”^15^. This is what we seek to analyze and discuss in the reforms of health systems conducted in Latin America and the Caribbean.

## METHODOS

We conducted a comparative study of the health systems of six selected countries in Latin America and the Caribbean, seeking similarities and structural differences in the implementation of the recommendations of international organizations, most notably the WB^17^, for reforms in health systems in the 1990s. The main objectives for the reforms were listed and their relationship with the neoliberal guidelines linked to the Washington Consensus stood out. The comparisons sought similarities, differences or associations among contemporary phenomena that occurred (or not) in different spaces to better understand them^18^. The analysis systematized similarities and differences between the reforms recommended and implemented in the studied countries.

The selected countries were the ones with the largest population and those for which there were the largest number of publications discussing their reforms from the 1990s: Brazil, Mexico, Colombia, Peru, Argentina and Chile. Venezuela has been excluded because its recent structural problems could jeopardize the results.

The categories of analysis were elaborated based on the WB’s guidelines for health system reforms, published in 1993 (Investing in Health*)*^17^, plus the synthesis of reform and the typologies of systems according to Mesa-Lago^19^ and Cruces^20^. These categories were transformed into characteristics to be analyzed in each health system, namely: co-payment, privatization mechanisms, decentralization, fragmentation of the system, integration of funding sources and coverage of the population (universal or segmented). These characteristics were described and analyzed to infer about the pertinence of the idea of Dadot and Laval^15^ of business logic in the public action of the State.

The documentary research was based on reports published by the Economic Commission for Latin America and the Caribbean (ECLAC), Pan American Health Organization (PAHO) and WB, publications of the Oswaldo Cruz Foundation and scientific articles from SciELO, PubMed and Google Scholar databases, based on the following combinations of terms: “neoliberalism,” “Latin America,” “health systems” and “reforms,” in English, Portuguese and Spanish, published in the period 2000 to 2017.

## RESULTS

The effects of neoliberalism on health systems were identified in the documents and publications analyzed according to BM’s recommendations^17^: a) reduction in the State’s responsibility in the financing of health services that benefited few, to make public resources available to those that benefited society in general – vaccines, control of vector transmission diseases, waste treatment, among others; b) imposition of burdens on users of public health services, especially those of curative care; (c) promoting risk coverage programs; d) strengthening the provision of health services by non-governmental institutions and stimulating the private market and competition among services; e) decentralization of the public health system, with greater financial and administrative autonomy to local governments, which would assume greater responsibility in the planning, budgeting and execution of public health activities; f) creation of an early payment system for those with an employment contract, such as mandatory health insurance, to increase competition among insurers and reduce administrative costs. In this document, entirely intended for health, the WB^17^ lists four problems to attack in health systems: poor allocation of resources, with the spending of public money on health interventions of “low cost-effectiveness*”*; inequality, such as lack of access for the poor to basic health services and low-quality services; inefficiency, such as wasted money funding drug brands rather than generic drugs, poorly mobilized and supervised health workers, underutilized hospitals; and explosive health costs, growing more than rents.

Table 1 summarises the specific characteristics that have been introduced in the reforms of the selected countries to address these recommendations. Chart 2 shows how the characteristics of health systems, described at the country level in Chart 1, seek to meet the principles of cost-reduction economic efficiency and increased competition to obtain it, much more than broad access to high-quality health services.

**Table:** Chart 2. Meanings and effects of health system reforms.

	Meaning	Effects
Co-payment	Mechanism, in which it is mandatory for the patient/insured/user to bear part of the costs of health services at the time of use. This mechanism of mandatory direct participation in costs has other denominations, such as moderating rate, participation in costs (cost-sharing), co-participation or user counterpart^27^. Fixed rate imposition for each medical service, introduction of a variable rate representing a percentage of the total cost of a service, combinations of fixed amounts and percentage rates or “annual deductible” system, i.e. setting a minimum annual level for medicine or service expenses per patient, below which no reimbursement is granted. System mainly used in private insurance.	State cost reduction: Transfer costs to the user. Increasing inequality: Increased *out-of-pocket* spending, leading to reduced access to promotion and prevention measures; worsening in treatment adherence; waiver or postponement of the use of services, especially by the older adults, chronically ill and low-income people; and increasing social inequalities. Cost increase in the medium and long term: Additional administrative expenses and higher subsequent costs with less health.
Privatization mechanisms	Three main mechanisms: promotion of the State for the expansion of the private sector (purchase of private services by the State, encouraging the participation of private entities in the management of resources and provision of services), tax exemption for users of health plans and private services.	State cost reduction: Transfer of costs to the private sector and increased competition. Increasing inequality: Provision of differentiated services related to access and quality. Cost increase in the medium and long term: Increased costs of high technology or deprivation of those that cannot afford access to available technology.
Decentralization	Subnational or local governments assumed greater responsibility with the planning, budgeting and execution of public health activities.	Central government cost reduction: Transfer of costs from national governments to subnational and local ones. Although it reduces the distance between the population and immediate managers, expanding the pressure power of users, it reduces the responsibility of national governments and their financing. Increasing inequality: Differentiated treatments in terms of quality and availability, according to different possibilities among regions and localities. Cost increase: Loss of economies of scale in purchases and public procurement.
Segmentação	Subsystems with different modalities of financing, affiliation and provision, each of them “specialized” in different strata of the population, according to their labor insertion, level of entry, ability to pay and social class. One or more public entities coexist, social insurance and several funders, guarators and private providers.	State cost reduction: Transfer of costs to the various funders. Increasing inequality: Worsening of the inequality in access and quality of services between different population groups.
Fragmentation	Coexistence of non-networked units and services or establishments that do not mutually cooperate, ignore and/or compete with other providers. Multiple agents operating without integration prevent the standardization of content, quality and costs of service provision. Generates increases in transaction costs and inefficient allocation of system resources.	Cost reduction: Exemption of the State from the provision of the public service. Competition among service providers. Increasing inequality: Reduction in universal access to the health service, lack of coordination increases the risk of some segments of the population being discovered, loss of solidarity of the system accentuates segregation of groups of the population and inequalities in access and use of services. Cost increase: Inefficient transaction and allocation costs generate larger resources.

**Chart 1:** Characteristics of health systems derived from recommendations based on the Washington Consensus19–23,27,30.

Co-payment	Privatization mechanisms	Decentralization	System fragmentation	Integration of funding sources	Population coverage (universal or segmented)
ARGENTINA					
No	Yes It incorporated competition among entities through the choice of workers between social work and private insurance and stimulated the expansion of prepaid medicine companies.	High decree High degree of decentralization for 24 provinces and some municipalities.	Tripartite Low coordination among the three subsectors: social insurance (which covers most of the population), the public sector (in charge of the provinces) and the private sector, each with its own financing and provision of services with universal coverage.	Low Low or zero integration of general incomes and social security contributions.	Segmented Social security for workers’ health is operated by social works and charges 6% of workers’ wages (6%) and employers (3%) by payroll. Includes domestic workers, pensioners and dependents (children and spouse). It does not include informal or self-employed.
BRAZIL					
No	Yes It stimulates privatization through the purchase of more complex services from the private sector, which has the largest number of hospital beds, in addition to exemption for users of health plans and private services.	High decree Very high degree of decentralization: federal government, 27 states and 5,507 municipalities (90% of them control primary care).	Dual Some coordination between the public subsector divided into federal, state and municipal levels (financing and provision functions) and the complementary private subsector.	High General rents and integrated systems from non-contributory financing.	Universal It includes all formal, domestic and agricultural workers, pensioners and dependents (children and spouse) and informal or self-employed workers.
CHILE					
Yes	Yes It stimulated the privatization of the assurance and boosted private medical care.	High decree High degree of decentralization: 28 regions and 342 communes (municipalities).	Dual Coordinated, combining the public (social insurance) and private subsectors, with separate financing and provision functions (this majority of the public subsector, i.e. universal insurance).	High Low or zero integration of general incomes and social security contributions.	Universal for PHC and segmented for curative care It preserved the choice by formal workers between contributing 7% of salaries to private insurance (Isapres) or public insurance (Fonasa) through social contributions. Includes domestic workers, pensioners and dependents (children and spouse). Excludes informal or self-employed ones.
COLOMBIA					
Yes	Yes It stimulated privatization by promoting the participation of the private sector in the administration of social insurance resources and in the provision of health services.	High decree High degree of decentralization: 32 departments and 524 municipalities (not complete).	Quadripartite Coordinated, with a public subsector (social insurance, divided into contributory and non-contributory regime), a private subsector and a public (linked) subsector.	High Low or zero integration of general incomes and social security contributions.	Universal for PHC in implementation The Mandatory Health Plan was created (POS), consisting of a single package of health services for each individual. Includes domestic workers, pensioners and dependents (children and spouse). It does not include informal or self-employed and agricultural workers.
PERU					
Yes	Yes The *Aseguramiento Universal en Salud* (AUS), created through Law No. 29,344 2009 and implemented in the same year by a new *Plan Esencial de Salud* (PEAS –Basic Health Plan). The law established mandatory insurance and free access to health care residents of the country through the PEAS. The plan also determined the dissociation of safe functions and provision of health services, promoting the participation of private entities in the health system.	Low degree Low degree of decentralization, from central government to 24 departments (20% in 2001); new plan decentralisation in 2005.	Tripartite: Public, social and private insurance, lacking adequate coordination among the three subsectors, without or with low separation of functions.	Low Low or zero integration of general incomes and social security contributions.	Segmented The public sector, through the ministry of health and integral health insurance (SIS) network, predominantly serves the poor population that that is uncovered by a health insurance (about 54%). EsSalud serves formal workers, for optional individual insurance or collective insurance (made by the employer), covering 7 to 11 million people. It offers both services of high complexity and primary care. It includes all formal, domestic and agricultural workers, pensioners and dependents (children and spouse) and informal or self-employed workers.
MEXICO					
Yes	Yes Purchase of services by the public sector and incentive to hire private (still limited).	Median degree From the federal government to all states, little for municipalities; decentralization	Tripartite: social and private insurance, segmented without coordination.	Low Low or zero integration of general incomes and social security contributions.	Segmented In 2003, they created popular insurance with insufficient federal funding and a restricted package of services. It includes all formal, domestic and agricultural workers, pensioners and dependents (children and spouse) and informal or self-employed workers.

PHC: primary health care

### Co-payment

According to Chart 1, four countries have instituted co-payment mechanisms, in which it is mandatory for the patient/insured/user to bear part of the costs of health services at the time of use. This mechanism of mandatory direct participation in costs presents other denominations, such as moderating rate, participation in costs (cost-sharing), co-participation or user counterpart^21-22^. Fixed rate imposition for each medical service, introduction of a variable rate representing a percentage of the total cost of a service, combinations of fixed amounts and percentage rates or “annual deductible” system, i.e. setting a minimum annual level for medicine or service expenses per patient, below which no reimbursement is granted.

The objectives of this measure are the recovery of costs of medical care, additional financial contribution, control of the allegedly exaggerated demand for medical services, limitation of unnecessary use, and, thus, control of the “moral risk” of users^22^. Increased out-of-pocket spending, leading to reduced access to promotion and prevention measures, worsening of treatment adherence, waiver or postponement of the use of services, especially by the older adults, chronically ill and low-income people; and increasing social inequalities. Patients give up necessary services and waive timely care, raising later care costs.

### Privatization

All the countries analyzed stimulated the privatization of health systems by three main mechanisms: promotion of the State to the expansion of the private sector (purchase of private services by the State, encouraging the participation of private entities in the management of resources and provision of services), tax exemption for users of health plans and private services. Competition between entities and the separation of functions are principles of the reform. All the countries analyzed were adherent to this guideline, which ends up transferring costs from the State to the private sector, but creates an impediment of access for those that cannot afford the private health costs^19^.

### Decentralization

The decentralization of public health systems, with greater financial and administrative autonomy of local governments, was adopted by all countries in different degrees^19^. Subnational or local governments assumed greater responsibility with the planning, budgeting and execution of public health activities. In practice, this strategy produced different effects: on the one hand, it decreased the distance between the population and immediate managers, increasing the pressure power of users; on the other hand, it represented a reduction in the responsibility of national governments, leading to a decrease in their funding. In the latter case, the poorest regions are punished, as a rule, with a harmful distributive result^24,8^.

### Integration, Fragmentation and Segmentation of Health Systems

Regarding the structure of health systems, they were analyzed for the degree of segmentation^19^. The segmented systems are characterized by having different sources of financing and by stratification of the population according to their labor insertion, income level, ability to pay and social class. Coexistence of non-networked units and services or establishments that do not mutually cooperate, ignore and/or compete with other providers. As a consequence, segmented and fragmented systems consolidate and worsen the inequalities in access and use of services among different population groups, making it difficult to standardize them in relation to costs and quality, among others^11^. The most fragmented systems were the quadripartite (four countries) and tripartite (three countries). The least were the fragmented dual (two countries).

In the sources of funding (general taxes, social security contributions and private spending), the most supportive systems “are those in which the entire population is covered by a system financed by general taxes”^32^. Less supportive systems occur when there is higher private spending (such as “out of pocket” or insurance payment), because access is tied to the user’s ability to pay, resulting in more iniquity^25^.

The integration of funding sources was low in three countries. The larger the number of funders, the more fragmented the system and the more difficult the coordination among them, increasing the risk of some segments of the population being discovered. Fragmentation dishonors the State’s responsibility to provide the public service, since it reduces the universal access, unlike when it is the sole funder^19^.

### Population Coverage

Population coverage of public health services was divided into universal, when there is no distinction by groups, and segmented, when there is a distinction of population groups according to the type and value of the contribution or work bond, or when there are specific service networks for each segment. Countries that have segmented coverage separate formal, domestic and agricultural workers, pensioners and dependents (children and spouse) and, for the most part, do not include informal or self-employed workers. The number of informal workers in the region is expressive and, in this condition, resulting in absence of health protection^11,19^.

The more segmented the system, the greater the probability of differentiated care, in terms of availability and quality of the services provided. The increase in the quality of service with the increased competition is the main argument of those defending segmentation. However, this argument ignores that the lower-income segment has, as a general rule, little knowledge and power of pressure, as well as little weight in the formation of public opinion, which tends to revert to worsening of the quality of the services provided. Only Brazil has universal access and, despite being provided for in legislation, it is not a practice observed in official statistics.

## DISCUSSION

The analysis of the reforms of the health systems of the LAC countries showed different degrees of adherence to the guidelines of multilateral organizations. In common, there was a search for efficiency and reduction in public spending, in addition to reducing the participation of national governments through decentralization and fragmentation of health systems, with segmentation by coverage groups, according to income and labor bond.

The comparative analysis signals how the reforms adapted to the change in the role of the State that characterized neoliberalism in the conception of Dardot and Laval^15^. More specifically, we tried to show “the transformation of public action, making the State a sphere that is also governed by competition rules and subjected to efficacy requirements similar to those that subject private companies” (p. 272) in health reforms. Thus, more than verifying the success or failure of the objectives of the WB’s reform proposals, as analyzed in Homedes and Ugalde^28^, or proposing alternatives for achieving these objectives, such as in Londoño and Frenk^29^, this article sought traces of such transformation of public action in the neoliberal direction.

In the political devices and strategies adopted for adjustments in health systems (Chart 2), we observed that a notion of economic cost to be reduced in the short term and a proposal to reduce inequality prevailed, with a sense of guaranteeing poor basic health services to the poor. The decentralization, implemented in most countries, aimed to free up funds from the central government to pay for the enormous public debt, shifting the financial burden of the social protection system, notably health, to subnational governments, which resulted in a quick implementation^28^. The positive effects alleged, such as participatory management, decisions based on local needs and improvement of the quality of service provision, were rarely seen^28^.

Co-payment and privatization divide state costs with the private initiative of patients or service providers. However, family out-of-pocket spending increases, leading to the abdication of spending on prevention, renunciation or postponement of health services, increasing inequality among patients, which may increase future care spending.

The fragmentation of systems, with the coexistence of non-networked service units, leads to ignorance and competition of the participants of the services offered by other providers, preventing the standardization of content and product quality. It increases inequality in the provision of health services and inefficient allocation with doubling costs. The more fragmented the system, the more difficult to coordinate its parts, and the greater the risk that a portion of the population to become unassisted.

The segmentation of the population, with division between those that contribute and those that do not, represented a strong focus of policies – seeking to reconcile basic services to the poor with cost reduction – and occurred in favor of universal coverage^29^. The segmentation of supply in most countries occurred according to the social classes and the type of protection guaranteed by the different modalities of public or private insurance, with the creation of “baskets” of basic care, according to the ability of the user to pay^28^. This produces greater inequality in the quality of health care received by the population^11,31^. Thus, although it is not the objective of this article to analyze the effects of reforms on reducing inequality, we can affirm that they tended to increase inequality from a qualitative point of view and to maintain privileges of population groups through population segmentation^29^.

The result is that many of the LAC countries maintain great asymmetries in access to the care system. This deepens internal inequalities and threatens the sustainability of universal health systems in their ability to protect a population immersed in multidimensional inequality. The mobilization in favour of universal health systems is also urgent, because the equality of the system forces the whole society to defend it. In the words of Felix Rígoli^32^, “the lesson of recent history tells us that there is a virtuous effect of the universalization of health and education services, not only to reduce poverty and inequality, but also to promote social cohesion and democracy.”

## CONCLUSIONS

The reforms of health systems in Latin America have met the objective of reducing costs, expanding competition among health providers and undergoing the neoliberal prescription, which leads the State to business-type behavior. The economic cost to the State was reduced, in particular that of the central governments responsible for public debt, whose payment was the immediate objective of the BM’s recommendations. Paradoxically, the uncoverage of part of the population may increase these costs in the future, due to the worsening of health problems of those unassisted due to payment difficulties.

The desired equality is questionable in the very design of the proposed reform measures. The differentiated treatment of the population leaves part of it uncovered by health services. The weight of opinion formers is reduced due to the division of the access to health by income classes, which would ensure greater availability and quality of health services. Therefore, coping with the effects of neoliberal reforms on health, which occurred in the 1990s, requires a blunt and multidimensional counter-reform that makes health a right of all again and seeks a solidarity system that leads to the reduction in inequalities and a more democratic society. This implies resuming principles such as solidarity (everyone pays and everyone uses), social participation, universal access and comprehensive care in the original sense of social protection of populations in all their needs.
