# Supplementary material for: Neoliberalism in Latin America: effects on health system reforms
Source: Rev Saude Publica. 2020 Jul 20;54:74. doi: 10.11606/s1518-8787.2020054001806 (PMC7371409; doi:10.11606/s1518-8787.2020054001806)
Supplement: Translation

# Translation: Neoliberalismo na América Latina: efeitos nas reformas dos sistemas de saúde

**Authors:** Leila Bernarda Donato Göttems, Maria de Lourdes Rollemberg Mollo

**Affiliations:** I Brasil Universidade de Brasília. Faculdade de Economia, Administração, Contabilidade e Gestão de Políticas Públicas. Programa de Pós-Graduação em Economia. Brasília, DF, Brasil

**Keywords:** Sistemas de Saúde, Reforma dos Serviços de Saúde, Política de Saúde, América Latina

## Abstract

**OBJETIVO:**

Analisar as recomendações dos organismos internacionais pautadas no Consenso de Washington para as reformas dos sistemas de saúde de países selecionados da América Latina e Caribe nas décadas de 1980 e 1990 e investigar os efeitos da lógica concorrencial de mercado sobre a ação pública nos sistema de saúde.

**MÉTODOS:**

Análise comparada de características das reformas dos sistemas de saúde realizadas nas décadas de 1980 e 1990, presentes até o momento no Brasil, Argentina, Chile, Colômbia, México e Peru. Os dados foram coletados por análise documental e revisão de literatura. Os sistemas foram descritos quanto às características de: copagamento, mecanismos de privatização, descentralização, fragmentação do sistema, integração das fontes de financiamento e cobertura da população (universal ou segmentada).

**RESULTADOS:**

As reformas foram implementadas de forma variada, aprofundando desigualdades nos sistemas de prestação de serviços de saúde. As mudanças, ao serem relacionadas à ideia neoliberal de transformar a ação pública na direção da lógica privada, apontam para o predomínio das regras da concorrência e da redução dos custos econômicos em todos os países analisados, contrariando a lógica dos sistemas universais de saúde.

**CONCLUSÃO:**

A redução dos custos econômicos, a fragmentação dos sistemas e as desigualdades na prestação de serviços de saúde, entre outros, poderão significar outros custos futuros decorrentes da baixa proteção à saúde da população. É imprescindível uma contrarreforma contundente e multidimensional, que retome a saúde como direito de todos, em um sistema solidário que possa levar à redução das desigualdades e a uma sociedade mais democrática.

## INTRODUÇÃO

Este artigo analisa os efeitos das recomendações neoliberais de organismos internacionais sobre o desenvolvimento de sistemas de saúde da América Latina e Caribe (ALC). Tais recomendações caracterizaram o neoliberalismo no mundo todo, desde o final da década de 1970, mas na ALC isso se verificou a partir dos 1980, com exceção do Chile, cuja liberalização se iniciou com a ditadura de Pinochet nos anos 1970.

Trata-se do período da chamada “crise da dívida”, decorrente do aumento, em 1979, das taxas de juros dos EUA. Isso levou os países da ALC à inadimplência em empréstimos, começando com o México, espalhando uma onda de choque financeiro em todo o continente^1^. Em outubro de 1983, 27 países de renda baixa e média, muitos nas Américas, estavam inadimplentes em seus empréstimos ou em processo de reescalonamento de dívidas^3,6^.

O Fundo Monetário Internacional (FMI) e o Banco Mundial (BM), nesse período, forneceram empréstimos aos países devedores para aliviar os déficits na balança de pagamentos e o ônus do serviço da dívida, bem como para socorrer o setor bancário privado em países de alta renda^6,8^. Associadas a esses empréstimos, haviam condicionalidades que eram medidas econômicas destinadas a abrir os mercados domésticos à penetração estrangeira e estimular as exportações de baixo custo. Essas recomendações baseavam-se no chamado Consenso de Washington, que continha dez medidas, desde disciplina fiscal, redução de gastos públicos, reforma fiscal e tributária (aumentando a base de tributação e tributando principalmente de forma indireta), até abertura comercial e econômica dos países, liberalização da taxa de câmbio e do comércio exterior, eliminação de restrições ao investimento financeiro direto, privatização e venda de estatais, desregulamentação e direito à propriedade intelectual^1^. Essas medidas levavam à redução do Estado de bem-estar social e à globalização dos interesses do capital, adotadas primeiramente na Grã-Bretanha de Margaret Thatcher e nos EUA de Ronald Reagan, e, com a crise da dívida, se alastraram rapidamente para as economias do Terceiro Mundo, como a ALC^1^.

Na América Latina se desenvolveu um tipo de capitalismo tardio com processo de urbanização acelerado e desordenado e incremento de atividades informais, nos quais são abundantes trabalhos servis e impregnados por relações de subordinação e baixa proteção^10^. A implantação do Estado de bem-estar social foi tardia, incompleta e bastante desigual entre os países. Esse fato, associado à frágil cidadania da população, fizeram com que, no momento da crise da dívida, as “reformas” agravassem a segmentação institucional e a fragmentação operacional dos sistemas de proteção social, ampliando as desigualdades de cobertura e acesso dos sistemas de saúde da região^11^.

As mudanças geraram reconfiguração dos sistemas de saúde e foram adotadas de forma desigual e gradual pelos países da ALC nos anos 1980 e 1990. Elas se basearam na separação entre política social e econômica, combinando programas de compensação focalizados no curto prazo, com a confiança renovada no crescimento econômico e no efeito derrame no longo prazo, relegando o conflito em torno da produção e apropriação da riqueza para segundo plano^6^. Na primeira década do século XXI, no período conhecido como “maré rosa” da ALC, muitos países lograram êxito na redução da extrema pobreza e nas desigualdades sociais, com aumento de renda e empregos formais, ajustes fiscais, aumento dos gastos sociais e reformas tributárias^11^. Todavia, não se conseguiu desenvolver um sistema de proteção social baseado no direito de acesso universal, e parte dele sofre atualmente os efeitos de um ressurgimento agudo do neoliberalismo.

As origens do neoliberalismo remontam à criação de um arcabouço teórico de regulação da vida na sociedade, do indivíduo e do Estado de tal forma a se contrapor aos estados planificadores e com poderes de interferir na liberdade individual, no direito de propriedade e nas livres trocas no mercado. Baseia-se no uso sistemático do poder do Estado, sob a aparência ideológica da “não intervenção”, para impor um projeto hegemônico de recomposição da regra do capital em cinco níveis: alocação de recursos domésticos, integração econômica internacional, reprodução do Estado, ideologia e reprodução da classe trabalhadora^7,9,13^.

O neoliberalismo provoca mudanças importantes na conformação das políticas sociais, principalmente decorrentes da reconfiguração dos Estados com a desregulamentação e privatizações, que os retiram de várias áreas e reduzem sua atuação^7,9,13^. A nova norma mundial exige que os dispositivos administrativos e sociais custem menos e se orientem para as exigências da competição econômica. Emergem nas políticas públicas os temas como a eficiência da gestão e novos métodos para fornecer serviços à população. Esse pensamento alimenta a vontade de impor, no cerne da ação pública, valores, práticas e funcionamento de empresa privada que conduzem à instituição de uma nova prática de governo – Estado flexível, reativo, fundamentado no mercado e orientado ao consumidor –, o *management*^15^. Assim, a lógica de eficiência é a econômica, que prioriza a redução de custos, a noção de igualdade no recebimento do serviço de saúde procura garantir o básico de forma focalizada e a norma de conduta não apenas da empresa, mas do indivíduo e do Estado, passa a ser a norma da concorrência, típica do mercado^6,8,12,15^.

O novo gênero de política social consiste então em enfraquecer o poder de negociação dos sindicatos, degradar o direito trabalhista, baixar o custo do trabalho, diminuir o valor das aposentadorias e a qualidade da proteção social, em nome da adequação à globalização^13^. Todavia, não é o fim do Estado, mas a relativização de seu papel como entidade integradora de todas as dimensões da vida coletiva, vez que tendem a delegar grande parte de suas funções às empresas privadas que, com frequência, já são globalizadas ou obedecem a normas mundiais. O Estado se põe a serviço de interesses oligopolistas específicos e não hesita em delegar a eles uma parte considerável da gestão sanitária, educacional, turística e lúdica da população. Em outras palavras, a regra geral no neoliberalismo é a concorrência e, com ela, a “compressão dos custos salariais e das despesas de proteção social dos Estados”^15^. É o que procuraremos analisar e discutir nas reformas dos sistemas de saúde realizadas na América Latina e Caribe.

## MÉTODO

Realizou-se um estudo comparado dos sistemas de saúde de seis países selecionados da América Latina e Caribe, buscando-se as semelhanças e diferenças estruturais na implantação das recomendações dos organismos internacionais, mais notadamente o BM^17^, para as reformas nos sistemas de saúde na década de 1990. Foram elencados os principais objetivos para as reformas e destacada sua relação com as diretrizes neoliberais ligadas ao Consenso de Washington. As comparações buscaram semelhanças, diferenças ou relações entre fenômenos contemporâneos ou não que ocorreram em distintos espaços para melhor compreendê-los^18^. A análise sistematizou semelhanças e diferenças entre as reformas recomendadas e implementadas nos diversos países.

Os países selecionados foram os de maior população e aqueles para os quais havia maior número de publicações discutindo suas reformas a partir dos anos 1990: Brasil, México, Colômbia, Peru, Argentina e Chile. Excluiu-se a Venezuela, cujos problemas estruturais dos últimos anos poderiam comprometer os resultados.

As categorias de análise foram elaboradas a partir das diretrizes do BM para as reformas dos sistemas de saúde, publicadas em 1993 (*Investing in Health)*^17^, acrescidas da síntese da reforma e das tipologias dos sistemas segundo Mesa-Lago^19^ e Cruces^20^. Essas categorias foram transformadas em características a serem analisadas em cada sistema de saúde, quais sejam: copagamento, mecanismos de privatização, descentralização, fragmentação do sistema, integração das fontes de financiamento e cobertura da população (universal ou segmentada). Tais características foram descritas e analisadas de forma a inferir sobre a pertinência da ideia de Dadot e Laval^15^ de lógica empresarial na ação pública do Estado.

A pesquisa documental baseou-se em relatórios publicados pela Comissão Econômica para a América Latina e o Caribe (Cepal), Organização Pan-Americana de Saúde (Opas) e BM, publicações da Fundação Oswaldo Cruz e artigos científicos localizados nas bases SciELO, PubMed e Google Acadêmico, a partir das seguintes combinações de termos: “neoliberalismo”, “América Latina”, “sistemas de saúde” e “reformas”, tanto no idioma inglês quanto português e espanhol, publicados no período de 2000 a 2017.

## RESULTADOS

Os efeitos do neoliberalismo sobre os sistemas de saúde foram identificados nos documentos e publicações analisados à luz das recomendações propostas pelo BM^17^: a) redução da responsabilidade do Estado no financiamento dos serviços de saúde que beneficiavam a poucos, para disponibilizar recursos públicos àqueles que beneficiavam a sociedade em geral – vacinas, controle de doenças de transmissão vetorial, tratamento de resíduos, entre outros; b) imposição de encargos aos usuários de serviços públicos de saúde, em especial àqueles da atenção curativa; c) fomento a programas de cobertura de riscos; d) fortalecimento da prestação de serviços de saúde por instituições não governamentais e estímulo ao mercado privado e à competição entre os serviços; e) descentralização do sistema público de saúde, com maior autonomia financeira e administrativa aos governos locais, que assumiriam maior responsabilidade no planejamento, orçamento e execução das atividades de saúde pública; f) criação de sistema de pagamento antecipado, como seguro obrigatório de saúde, para quem tem contrato de trabalho, para aumentar a competição entre seguradoras e reduzir custos administrativos. Nesse documento, inteiramente destinado à saúde, o BM^17^ elenca quatro problemas a atacar nos sistemas de saúde: má alocação de recursos, com os gastos de dinheiro público em intervenções de saúde de “*low cost-effectiveness*”; desigualdade, como falta de acesso dos pobres a serviços básicos de saúde e serviços de baixa qualidade; ineficiência, como dinheiro desperdiçado financiando marcas de medicamentos ao invés de drogas genéricas, trabalhadores na área de saúde mal mobilizados e supervisionados, hospitais subutilizados; e custos de saúde explosivos, crescendo mais que as rendas.

No Quadro 1 estão sintetizadas as características específicas que foram introduzidas nas reformas dos países selecionados, de forma a contemplar tais recomendações. O Quadro 2 descreve como as características dos sistemas de saúde, descritas em nível de país no Quadro 1, procuram atender aos princípios de eficiência econômica de redução de custos e ao aumento da concorrência para obtê-la, muito mais do que ao acesso amplo a serviços de saúde de alta qualidade.

**Quadro 1:** Características dos sistemas de saúde que são oriundas das recomendações baseadas no Consenso de Washington19–23,27,30.

Copagamento	Mecanismos de privatização	Descentralização	Fragmentação do sistema	Integração das fontes de financiamento	Cobertura da população (universal ou segmentada)
ARGENTINA					
**Não**	**Sim** Incorporou a competição entre entidades por meio da escolha dos trabalhadores entre obra social ou seguro privado e estimulou a expansão das empresas de medicina pré-paga.	**Alto grau** Alto grau de descentralização para 24 províncias e alguns municípios.	**Tripartite** com baixa coordenação entre os três subsetores: seguro social (que cobre a maioria da população), o setor público (a cargo das províncias) e o privado, cada um com seu próprio financiamento e provisão de serviços com cobertura universal.	**Baixa** Baixa ou nula integração de rendas gerais, cotizações e seguridade social.	**Segmentada** A seguridade social para a saúde dos trabalhadores é operada pelas obras sociais e cobra 6% dos salários dos trabalhadores (6%) e empregadores (3%) pela folha de pagamento. Inclui trabalhadores domésticos, agrícolas, pensionados e dependentes (filhos e cônjuge). Não inclui os informais ou por conta própria.
BRASIL					
**Não**	**Sim** Estimula a privatização por meio da compra de serviços de maior complexidade do setor privado, que dispõe da maior quantidade de leitos hospitalares, além de isenção fiscal a usuários de planos de saúde e serviços privados.	**Alto grau** Muito alto grau de descentralização entre governo federal, 27 estados e 5.507 municípios (90% deles controlam a atenção primária).	**Dual** com certa coordenação entre o subsetor público dividido em níveis federal, estatal e municipal (funções de financiamento e provisão) e o subsetor privado complementar.	**Alta** Rendas gerais e sistemas integrados a partir do financiamento não contributivo.	**Universal** Inclui todos os trabalhadores formais, domésticos, agrícolas, pensionados e dependentes (filhos e cônjuge) e os trabalhadores informais ou autônomos.
CHILE					
**Sim**	**Sim** Estimulou a privatização do asseguramento e impulsionou a atenção médica privada.	**Alto grau** Alto grau de descentralização: 28 regiões e 342 comunas (municípios).	**Dual** coordenado, combinando os subsetores público (seguro social) e privado, com funções separadas de financiamento e provisão (esta majoritária do subsetor público, ou seja, seguro **universal**).	**Alta** Integração de rendas gerais e cotizações de seguridade social.	**Universal para a APS e segmentada para a atenção curativa** Preservou a escolha pelos trabalhadores formais entre contribuir com 7% dos salários para seguros privados (Isapres) ou para o seguro público (Fonasa) por meio de contribuições sociais. A cobertura inclui trabalhadores formais, domésticos, agrícolas, pensionados e dependentes (filhos e cônjuge). Exclui os informais ou autônomos.
COLÔMBIA					
**Sim**	**Sim** Estimulou a privatização promovendo a participação do setor privado na administração dos recursos do seguro social e na prestação dos serviços de saúde.	**Alto grau** Alto grau de descentralização: 32 departamentos e 524 municípios (não completa).	**Quadripartite** coordenado, com um subsetor público (seguro social, dividido em regime contributivo e não contributivo), um subsetor privado e um de caráter público (vinculado).	**Alta** Integração de rendas gerais e cotizações da seguridade social.	**Universal para a APS em implementação** Foi criado o Plano Obrigatório de Saúde (POS), composto por um pacote único de serviços de saúde destinado a cada indivíduo. Livre escolha dos prestadores pelos usuários. Inclui trabalhadores domésticos, pensionados e dependentes (filhos e cônjuge). Não inclui os informais ou autônomos e os agrícolas.
PERU					
**Sim**	**Sim** O *Aseguramiento Universal en Salud* (AUS), criado mediante a Lei nº 29.344 de 2009 e implementado no mesmo ano por um novo *Plan Esencial de Salud* (PEAS). A lei estabeleceu a obrigatoriedade dos seguros e o acesso gratuito à saúde de todos os residentes no país mediante o PEAS. O plano também determinou a dissociação das funções seguro e provisão de serviços de saúde, fomentando a participação das entidades privadas no sistema de saúde.	**Baixo grau** Baixo grau de descentralização, do governo central para 24 departamentos (20% em 2001); novo plano de descentralização em 2005.	**Tripartite:** público, seguro social e privado, carente de coordenação adequada entre os três subsetores, sem ou com baixa separação de funções.	**Baixa** Baixa ou nula integração de rendas gerais e cotizações para a seguridade social.	**Segmentado** O setor público, por meio da rede própria do Ministério da Saúde e do Seguro Integral de Saúde (SIS), atende predominantemente à população pobre que não dispõe de seguro-saúde (cerca de 54%). O EsSalud atende aos trabalhadores formais, por seguros individuais opcionais ou por seguros coletivos (feitos pela instituição empregadora), cobrindo de 7 a 11 milhões de pessoas. Oferece serviços de alta complexidade e atenção básica. Inclui todos os trabalhadores formais, domésticos, agrícolas, pensionados e dependentes (filhos e cônjuge) e exclui os trabalhadores informais ou autônomos.
MÉXICO					
**Sim**	**Sim** Compra de serviços pelo setor público e incentivo à contratação de privados (ainda limitada).	**Grau mediano** Do governo federal até todos os estados, com muito pouco para municípios; desconcentração limitada no seguro social (IMSS), não em outros.	**Tripartite:** público, seguro social e privado, segmentado sem coordenação.	**Baixa** Baixa ou nula integração de rendas gerais e cotizações para a seguridade social.	**Segmentada** Em 2003 criaram o seguro popular com financiamento federal e insuficiente e com um pacote restrito de serviços. Inclui todos os trabalhadores formais, agrícolas, pensionados e dependentes (filhos e cônjuge) e exclui os trabalhadores informais ou autônomos e os domésticos.

APS: atenção primária à saúde

**Quadro 2:** Significados e efeitos das reformas dos sistemas de saúde.

	Significado	Efeitos
**Copagamento**	Mecanismo em que há obrigatoriedade de o paciente/segurado/usuário arcar com parte dos custos dos serviços de saúde no ato da utilização. Tal mecanismo de obrigatoriedade de participação direta nos custos apresenta outras denominações, como taxa moderadora, participação no custeio (*cost-sharing*), coparticipação ou ainda contrapartida do usuário^27^. Imposição de taxa fixa para cada serviço médico, introdução de taxa variável que represente porcentagem do custo total de um serviço, combinações de quantias fixas e taxas percentuais ou sistema de “franquia anual”, ou seja, a fixação de um nível mínimo anual para as despesas de medicamentos ou de serviços por paciente, abaixo do qual nenhum reembolso é concedido. Sistema utilizado principalmente em seguros privados.	**Redução de custos do Estado:** Transferência de custos para o usuário. **Aumento da desigualdade:** Aumento do gasto *out-of-pocket*, levando à redução do acesso a medidas de promoção e prevenção; piora na adesão ao tratamento; renúncia ou postergação do uso de serviços, em especial por idosos, doentes crônicos e pessoas de baixa renda; e aumento das desigualdades sociais. **Aumento de custos em médio e longo prazo:** Gastos administrativos adicionais e custos posteriores maiores com menos saúde.
**Mecanismos de privatização**	Três principais mecanismos: fomento do Estado à expansão do setor privado, compra de serviços privados pelo Estado, estímulo à participação das entidades privadas na gestão de recursos e provisão de serviços, isenção fiscal a usuários de planos de saúde e serviços privados.	**Redução de custos do Estado:** Transferência de custos para a iniciativa privada e aumento da concorrência. **Aumento da desigualdade:** Prestação de serviços diferenciados em termos de acesso e qualidade. **Aumento de custos em médio e longo prazo:** Aumento dos custos com a alta tecnologia ou privação dos que não podem pagar ao acesso à tecnologia disponível.
**Descentralização**	Os governos subnacionais ou locais passaram a assumir maior responsabilidade com o planejamento, orçamento e execução das atividades de saúde pública.	**Redução de custos do governo central:** Transferência de custos dos governos nacionais para os subnacionais e locais. Embora diminua as distâncias entre a população e os gestores imediatos, ampliando o poder de pressão dos usuários, reduz a responsabilidade dos governos nacionais e seu financiamento. **Aumento da desigualdade:** Tratamentos diferenciados em termos de qualidade e disponibilidade, conforme possibilidades distintas entre regiões e localidades. **Aumento de custos:** Perda de economias de escala nas compras e nos contratos públicos.
**Segmentação**	Subsistemas com distintas modalidades de financiamento, afiliação e provisão, cada um deles “especializado” em diferentes estratos da população, conforme sua inserção laboral, nível de ingresso, capacidade de pagamento e classe social. Coexistem uma ou várias entidades públicas, o seguro social e diversos financiadores, asseguradores e prestadores privados.	**Redução de custos do Estado:** Transferência de custos para os diversos financiadores. **Aumento da desigualdade:** Aprofundamento da desigualdade no acesso e na qualidade dos serviços entre os diferentes grupos populacionais.
**Fragmentação**	Coexistência de unidades e serviços não integrados em rede de serviços ou estabelecimentos que não colaboram mutuamente, ignoram e/ou competem com os outros prestadores. Múltiplos agentes operando sem integração impedem a padronização dos conteúdos, da qualidade e dos custos da provisão de serviços. Gera incrementos nos custos de transação e alocação ineficiente dos recursos do sistema.	**Redução de custos:** Desoneração do Estado da prestação do serviço público. Concorrência entre prestadores de serviços. **Aumento da desigualdade:** Redução do acesso universal ao serviço de saúde; falta de coordenação aumenta risco de ficarem descobertos alguns segmentos da população; perda da solidariedade do sistema acentua segregação de grupos da população e desigualdades de acesso e utilização dos serviços. **Aumento de custos:** Custos de transação e de alocação ineficiente geram recursos maiores.

### Copagamento

Conforme o Quadro 1, quatro países instituíram mecanismos de copagamento. Trata-se de um procedimento em que há obrigatoriedade de o paciente/segurado/usuário arcar com parte dos custos dos serviços de saúde no ato da utilização. Tal mecanismo de obrigatoriedade de participação direta nos custos apresenta outras denominações, como taxa moderadora, participação no custeio (*cost-sharing*), coparticipação ou ainda contrapartida do usuário^21-22^. O copagamento pode ser aplicado de diferentes formas: imposição de taxa fixa para cada serviço médico, introdução de taxa variável que represente porcentagem do custo total de um serviço, combinações de quantias fixas e taxas percentuais ou sistema de “franquia anual”, ou seja, a fixação de um nível mínimo anual para as despesas de medicamentos ou de serviços por paciente, abaixo do qual nenhum reembolso é concedido, sistema utilizado principalmente em seguros privados^22^.

Os objetivos dessa medida são a recuperação de custos da atenção médica, aporte financeiro adicional, controle da demanda pretensamente exagerada por serviços médicos, limitação do uso desnecessário, e, com isso, controle do “risco moral” dos usuários^22^. Os resultados, contudo, podem evidenciar efeitos deletérios importantes: redução do acesso a medidas de promoção e prevenção; piora na adesão ao tratamento, renúncia ou postergação do uso de serviços, em especial por idosos, doentes crônicos e pessoas de baixa renda; gastos administrativos adicionais; e aumento das desigualdades sociais. Os pacientes abdicam de serviços necessários e renunciam à atenção em tempo oportuno, elevando custos assistenciais posteriores.

### Privatização

Todos os países analisados estimularam, de diferentes formas, a privatização dos sistemas de saúde. O fenômeno ocorreu por variados mecanismos: fomento do Estado à expansão do setor privado, compra de serviços privados pelo Estado e estímulo à participação das entidades privadas na gestão de recursos e provisão de serviços, além de isenção fiscal aos usuários de planos de saúde e serviços privados^25^. A competição entre entidades e a separação de funções serão princípios da reforma. Todos os países analisados foram aderentes a essa diretriz, que acaba por transferir custos do Estado para a iniciativa privada, mas cria um impedimento de acesso para os que não podem arcar com os custos privados da saúde^19^.

### Descentralização

A descentralização dos sistemas públicos de saúde, com maior autonomia financeira e administrativa dos governos locais, foi adotada por todos os países, em diferentes graus^19^. Os governos locais passaram a assumir maior responsabilidade com o planejamento, orçamento e execução das atividades de saúde pública. Na prática, essa estratégia produziu diferentes efeitos: por um lado, diminuiu as distâncias entre a população e os gestores imediatos, aumentando o poder de pressão dos usuários; por outro, representou uma redução da responsabilidade dos governos nacionais, levando à diminuição do seu financiamento. Nesse último caso, punem-se, em particular, as regiões mais pobres, via de regra mais necessitadas, com resultado distributivo danoso^24,28^.

### Integração, Fragmentação e Segmentação dos Sistemas de Saúde

Em relação à estrutura dos sistemas de saúde, eles foram analisados quanto ao grau de segmentação^19^. Os sistemas segmentados se caracterizam por ter distintas fontes de financiamento e por estratificar a população conforme sua inserção laboral, nível de renda, capacidade de pagamento e classe social. A fragmentação se desvela na coexistência de unidades e serviços não integrados em rede, que não colaboram mutuamente e que ignoram e/ou competem entre si e com outros prestadores. Como consequência, os sistemas segmentados e fragmentados consolidam e aprofundam as desigualdades de acesso e de utilização de serviços entre os diferentes grupos populacionais, dificultando a padronização deles em relação a custos e qualidade, entre outros^11^. Os sistemas mais fragmentados foram os quadripartites (um país) e tripartites (três países). Os menos foram os duais fragmentados (dois países).

Nas fontes de financiamento (impostos gerais, cotizações da seguridade social e gastos privados), os sistemas mais solidários “são aqueles em que toda a população é coberta por um sistema financiado por impostos gerais”^32^. Sistemas menos solidários ocorrem quando há maior gasto privado (do tipo “*out of pocket*” ou pagamento de seguros), porque o acesso está atrelado à capacidade de pagamento do usuário, traduzindo-se em mais iniquidade^25^.

Quanto à integração das fontes de financiamento, ela foi baixa em três países. Quanto maior o número de financiadores, diz-se que mais fragmentado é o sistema e mais difícil é a coordenação entre eles, aumentando o risco de ficarem descobertos alguns segmentos da população. A fragmentação desonera o Estado da responsabilidade de prestar o serviço público, reduzindo o acesso universal, ao contrário do que ocorre quando ele é o único financiador^19^.

### Cobertura da População

A cobertura populacional por serviços públicos de saúde foi dividida em universal, quando não há distinção por grupos, e segmentada, quando há distinção de grupos populacionais conforme o tipo e o valor da contribuição ou vínculo de trabalho, ou quando há redes de serviços específicos para cada segmento. Os países que possuem cobertura segmentada separam trabalhadores formais, domésticos, agrícolas, pensionados e dependentes (filhos e cônjuge) e, em sua maioria, não incluem os informais ou autônomos. A quantidade de trabalhadores informais na região éexpressiva e, nessa condição, eles estarão sem proteção de saúde^11,19^.

Quanto mais segmentado é o sistema, maior é a probabilidade de atendimentos diferenciados, em termos de disponibilidade e qualidade dos serviços prestados. O argumento de defesa da segmentação é de que há aumento da concorrência e isso ampliaria a qualidade dos serviços. O que esse argumento não deixa ver, todavia, é que o segmento de mais baixa renda tem, em regra, pouco conhecimento e poder de pressão e pouco peso na formação da opinião pública, o que tende a se reverter em piora da qualidade dos serviços prestados. Só o Brasil tem acesso universal e, embora isso esteja previsto na legislação, não é uma prática observada nas estatísticas oficiais.

## DISCUSSÃO

A análise das reformas dos sistemas de saúde dos países da ALC demonstrou que houve diferentes graus de adesão às diretrizes dos organismos multilaterais. Em comum, houve a busca pela eficiência e redução do gasto público, além de redução de participação dos governos nacionais por meio da descentralização e fragmentação dos sistemas de saúde, com segmentação por grupos de cobertura, conforme a renda e o vínculo trabalhista.

A análise comparativa sinaliza como as reformas se adequaram à mudança do papel do Estado que caracterizou o neoliberalismo na concepção de Dardot e Laval^15^. Mais especificamente, procurou-se mostrar como nas reformas da saúde observou-se “a transformação da ação pública, tornando o Estado uma esfera que também é regida por regras de concorrência e submetida a exigências de eficácia semelhantes àquelas que sujeitam as empresas privadas” (p. 272). Assim, mais do que verificar o sucesso ou insucesso dos objetivos das propostas de reformas do BM, como analisado em Homedes e Ugalde^28^, ou propor alternativas para a obtenção desses objetivos, como em Londoño e Frenk^29^, este artigo buscou traços de tal transformação da ação pública na direção neoliberal.

Percebe-se, nos dispositivos e estratégias políticas adotadas para os ajustes nos sistemas de saúde (Quadro 2), que predominou uma noção de custo econômico a ser reduzido em curto prazo e uma proposta de redução de desigualdade que tem o sentido de garantir aos pobres serviços básicos de saúde. A descentralização, implementada na maioria dos países, objetivou libertar fundos do governo central para pagar pelo enorme endividamento público, deslocando os encargos financeiros do sistema de proteção social, notadamente a saúde, para os governos subnacionais, o que resultou em uma maneira rápida de realizá-la^28^. Os efeitos positivos apregoados, tais como gestão participativa, decisões pautadas pelas necessidades locais e melhoria da qualidade da oferta de serviços, em muito poucas experiências se verificaram^28^.

O copagamento e a privatização dividem custos do Estado com a iniciativa privada dos pacientes ou das empresas prestadoras dos serviços. Contudo, aumentam os gastos familiares *out-of-pocket*, levando à abdicação de gastos de prevenção, renúncia ou postergação de serviços de saúde, aumentando a desigualdade entre os pacientes, o que pode aumentar os gastos assistenciais futuros.

A fragmentação dos sistemas, com coexistência de unidades de serviço não integrados em rede, leva à ignorância e concorrência dos participantes dos serviços oferecidos por outros prestadores, impedindo a padronização dos conteúdos e da qualidade dos produtos. Aumenta a desigualdade na prestação dos serviços de saúde e a alocação ineficiente com duplicação de custos. Quanto mais fragmentado é o sistema, mais difícil é a coordenação entre suas partes, e maior o risco de que uma parcela da população fique desassistida.

A segmentação da população, com divisão entre os que contribuem e os que não contribuem, representou forte focalização das políticas – procurando compatibilizar serviços básicos aos pobres com redução de custos – e ocorreu em desfavor da cobertura universal^29^. A segmentação da oferta na maioria dos países ocorreu de acordo com as classes sociais e o tipo de proteção garantido pelas diferentes modalidades de seguros públicos ou privados, com criação de “cestas” de cuidados básicos, conforme a capacidade de pagamento do usuário^28^. Isso produz maior desigualdade na qualidade da atenção à saúde recebida pela população^11,31^. Assim, ainda que não seja objetivo deste artigo analisar os efeitos das reformas quanto à redução da desigualdade, é possível afirmar que elas tenderam, via segmentação da população, a ampliar a desigualdade do ponto de vista qualitativo e a manter privilégios de grupos populacionais^29^.

O resultado é que muitos dos países da ALC mantêm grandes assimetrias no acesso ao sistema de cuidados. Isso aprofunda as desigualdades internas e ameaça a sustentabilidade dos sistemas universais de saúde em sua capacidade de proteger uma população imersa em desigualdade multidimensional. A mobilização em favor dos sistemas universais de saúde é também urgente, porque a igualdade do sistema para todos força a sociedade inteira a defendê-lo. Nas palavras de Felix Rígoli^32^, “a lição da história recente nos fala que há um efeito virtuoso da universalização dos serviços de saúde e educação, não apenas para reduzir a pobreza e a desigualdade, mas também para promover a coesão social e a democracia”.

## CONCLUSÕES

As reformas dos sistemas de saúde na América Latina obedeceram ao objetivo de redução de custos, do ponto de vista econômico, ampliando a concorrência entre prestadores de saúde e submetendo-se ao receituário neoliberal, que leva o Estado a um comportamento do tipo empresarial. O custo econômico para o Estado foi reduzido, em particular o dos governos centrais, responsáveis pela dívida pública, cujo pagamento era o objetivo imediato das recomendações do BM. Paradoxalmente, a desproteção de parte da população poderá ampliar tais custos no futuro, pelo agravamento dos problemas de saúde dos que ficaram sem prevenção e atenção por dificuldades de pagamento.

Quanto à igualdade desejada, é discutível na própria concepção das medidas propostas para as reformas. O tratamento diferenciado da população deixa parte dela descoberta com relação ao uso dos serviços de saúde. Ao dividir tal utilização por classes de renda, reduz-se o peso dos formadores de opinião, que garantiriam maior disponibilidade e qualidade dos serviços de saúde. O enfrentamento dos efeitos das reformas neoliberais na saúde, ocorridas nos anos 1990, requer, portanto, contrarreforma contundente e multidimensional, que retome a saúde como direito de todos e busque um sistema solidário que leve à redução das desigualdades e a uma sociedade mais democrática. Isso implica retomar princípios como os de solidariedade (todos pagam e todos usam), de participação social, de acesso universal e de atenção integral no sentido original de proteção social das populações em todas as suas necessidades.
